# Potentiating the Antitumor Activity of Cytotoxic T Cells *via* the Transmembrane Domain of IGSF4 That Increases TCR Avidity

**DOI:** 10.3389/fimmu.2020.591054

**Published:** 2021-02-01

**Authors:** Hye-Ran Kim, Jeong-Su Park, Yasmin Fatima, Maiza Kausar, Jin-Hwa Park, Chang-Duk Jun

**Affiliations:** ^1^School of Life Sciences, Gwangju Institute of Science and Technology (GIST), Gwangju, South Korea; ^2^Immune Synapse and Cell Therapy Research Center, Gwangju Institute of Science and Technology (GIST), Gwangju, South Korea

**Keywords:** immunoglobulin superfamily member 4, transmembrane, TCR complex, TCR avidity, immunotherapy

## Abstract

A robust T-cell response is an important component of sustained antitumor immunity. In this respect, the avidity of TCR in the antigen-targeting of tumors is crucial for the quality of the T-cell response. This study reports that the transmembrane (TM) domain of immunoglobulin superfamily member 4 (IGSF4) binds to the TM of the CD3 ζ-chain through an interaction between His177 and Asp36, which results in IGSF4-CD3 ζ dimers. IGSF4 also forms homo-dimers through the GxxVA motif in the TM domain, thereby constituting large TCR clusters. Overexpression of IGSF4 lacking the extracellular (IG4ΔEXT) domain potentiates the *OTI* CD8^+^ T cells to release IFN-γ and TNF-α and to kill OVA^+^-B16F10 melanoma cells. In animal models, IG4ΔEXT significantly reduces B16F10 tumor metastasis as well as tumor growth. Collectively, the results indicate that the TM domain of IGSF4 can regulate TCR avidity, and they further demonstrate that TCR avidity regulation is critical for improving the antitumor activity of cytotoxic T cells.

## Introduction

Although chimeric antigen receptor (CAR)-engineered T cells have shown promising outcomes for the treatment of certain B-cell malignancies (1–4), the CAR approach is limited to only recognizing cell surface structures (1–4). In contrast, TCR can allow the recognition of an entire array of potential intracellular proteins, which are processed, delivered, and presented as peptide/major histocompatibility complexes (p-MHC) on the cell surface. Therefore, one approach for cancer immunotherapy is the reactivation of tumor-infiltrating lymphocytes (TILs), with the aim that these T cells directly kill tumor cells (5). However, the major obstacle in TIL-based immunotherapy is that most tumor antigens are self-originating and often non-mutated. In this respect, the affinity between the TCR and p-MHC is generally thought to play the most central role in antigen recognition (6, 7). Thus, the affinity modification of TCR has been considered as a tool for augmenting the antitumor response of redirected T cells (1, 8–11). However, therapeutic applications of affinity-modified TCRs have resulted in unexpected toxicity in clinical trials (12–14). For instance, patients with metastatic colorectal tumors received engineered T cells expressing high-affinity TCRs suffered severe colitis after treatment (15). Thus, it is uncertain whether higher-affinity TCRs can render adoptive cell therapy (ACT) more effective in mediating antitumor activity.

An interesting paradox is that, despite the TCR having a very low affinity for its p-MHC (1–50 μM) (16), T cells possess very high sensitivity to their antigens. Immunologists have tried to resolve this paradox by considering that the TCRs may be organized into pre-existing nanoscale structures, termed TCR nanoclusters (16–20). In agreement with this hypothesis, it has been demonstrated that more TCR nanoclusters are present on antigen-experienced or memory cells than naïve T cells before antigen stimulation, suggesting a basis of strong TCR signaling by pre-clustering (21) and further implying that the avidity, rather than the affinity, of TCRs may be a more important determinant of TCR signaling in a condition where the antigenicity is relatively poor. However, the mechanism favoring the existence of TCR clusters in antigen-experienced or memory T cells are largely unknown.

A previous report demonstrated that the TCR avidity of activated T cells for peptide-MHC complexes is 20- to 50-fold higher than that of resting T cells (22), suggesting that activation-induced changes in membrane composition are related to TCR avidity. Consistently, activated and naïve T cells have been distinguished by changes in membrane organization and redistribution of certain molecules in the plane of the plasma membrane (23–26). For instance, key TCR signaling molecules may be associated with lipid rafts, domains enriched in specific lipids and cholesterol (25). Tetraspanin-enriched microdomains (TEMs) are another type of membrane platform distinct from lipid rafts, which contribute to receptor clustering and local accumulation of adhesion molecules in the immunological synapse (18). In agreement with this, tetraspanin CD81 positively regulates immunological synapse organization through its association with the CD3 ζ-chain (27). This evidence strongly suggests the possibility that changes in membrane organization or control of the expression of certain molecules on the T cell surface can control TCR avidity. In this regard, it is interesting to note that enhanced avidity is due to an increased cross-linking of TCRs on activated T cells (22). We therefore questioned whether any of T cell surface molecules can induce TCR cross-linking or clustering, thereby enhancing TCR avidity.

In accordance with this concept, it has been reported that immunoglobulin superfamily member 4 (IGSF4) physically interacts with the CD3 ζ-chain and enhances TCR-mediated signal transduction (28). IGSF4-deficient mice obtained from gene trap (GT) technology showed impaired TCR-mediated thymocyte selection and maturation and T cell activation. Interestingly, we found that “domain swapping” of IGSF4 transmembrane (TM) to CD43—a molecule excluded from the central supramolecular activation cluster (c-SMAC), failed to localize at the c-SMAC in the immunological synapse, demonstrating that the TM domain may serve as a physical partner with the CD3 ζ-chain. However, the molecular details of how the IGSF4 TM domain interacts with the CD3 ζ-chain remains unclear.

In the present study, it was found that IGSF4 not only forms a homodimer through the TM domain but also binds to the CD3 ζ-chain, which results in IGSF4-CD3-ζ multi-assembly on the cell surface. Since research has yet to exemplify modulation of the size of TCR clusters with a single factor to enhance avidity, the present study questioned whether the TM domain of IGSF4 can increase the TCR nanoclusters, thereby enhancing TCR signaling through avidity regulation. Further, this study investigated whether TCR-avidity regulation by the IGSF4 TM domain potentiates the antitumor response of CD8^+^ T cells *in vitro* and *in vivo*. The present data strongly demonstrate that TCR-avidity control is an important factor for enhancing the antitumor response in ACT.

## Materials and Methods

### Reagents and Antibodies

Rabbit polyclonal anti-GFP (ab6556) and anti-Myc (ab39688) antibodies were purchased from Abcam (Cambridge, MA, USA). Mouse monoclonal anti-CD3 ϛ-chain (6B10.2) was purchased from Santa Cruz Biotechnology (Dallas, TX, USA). HRP-conjugated anti-mouse IgG and anti-rabbit IgG were purchased from Cell Signaling Technology (Danvers, MA, USA). Duo Set ELISA Kits for human IL-2, mouse IL-2, IFN-γ, TNF-α, mouse anti-p-CD3 ϛ (K25-407.69), and mouse CD3^+^ T cell enrichment columns were obtained from R&D systems (Minneapolis, MN, USA). OVA peptide fragments (323–339 and 257–264) were purchased from GenScript (San Diego, CA, USA). APC-CD69 (12-0691-82), APC-anti-CD62L, and Percp-Cy5.5-CD44 were purchased from eBioscience (San Diego, CA, USA). Hybridoma cell lines 145-2C11 (mouse anti-CD3; CRL-1975), PV1 (mouse anti-CD28; HB-12352), and OKT3 (human anti-CD3; CRL-8001) were purchased from ATCC (Manassas, VA, USA). Reverse transcription polymerase chain reaction (PCR) premix and restriction enzymes were purchased from Enzynomics (Daejeon, Korea). The plasmid DNA purification kit and WEST-ZOL Western Blot Detection Kit were purchased from iNtRON Biotechnology (Seongnam, Korea). PrimeSTAR HS DNA polymerase was purchased from Takara Bio Inc. (Shiga, Japan). Poly-L-Lysine (PLL) was purchased from Sigma-Aldrich (St. Louis, MO, USA). Small interfering RNA (siRNA)-targeting IGSF4 and scrambled siRNA were purchased from Thermo Fisher Scientific (Waltham, MA, USA). CellTrace™ Violet (CTV), Cell-Tracker CMFDA-green, CMRA-Orange, and Lipofectamine 2000 reagent were purchased from Invitrogen (Carlsbad, CA, USA). APC-conjugated H-2K^b^ OVA tetramer-SIIGFEKL and APC-conjugated TRP2 tetramer as a negative control were purchased from MBL (Nagoya, Japan). Anti-mouse MHC class I (H-2K^b^) and anti-TCRβ (H57-597) were purchased from Bio-X-Cell (West Lebanon, NH, USA).

### Cells

Jurkat T (TIB-152), HEK293T (CRL-1573), and B16F10 (CRL-6475) cell lines were purchased from ATCC. Adult leukemia cell lines, MT2, and MT4 were purchased from CellBank Australia (Westmead, NSW, Australia). The retroviral ecotrophic packaging cell line Platinum-E was purchased from Cell Biolabs (San Diego, CA, USA). Cells were maintained in RPMI-1640 or Dulbecco’s modified Eagle medium (Invitrogen) supplemented with 10% (v/v) fetal bovine serum (FBS; Invitrogen). A stable B16F10 cell line expressing membrane-bound OVA (B16F10-OVA) was produced by transient transfection with pCL-neo-mOVA (Addgene, Cambridge, MA) using Lipofectamine 2000 reagent (Invitrogen) and selected with G418 (InvivoGen; San Diego, CA, USA). Naïve CD3^+^ T cells were purified from mouse spleen and lymph nodes by negative selection using a T-cell enrichment column (R&D Systems). Naïve CD4^+^ and CD8^+^ T cells and CD11C^+^ dendritic cells (DCs) were purified from mouse spleen and lymph nodes by negative selection using an EasySep magnetic separation system (Stemcell Technologies; Vancouver, Canada). To generate mouse T-cell blasts, isolated T cells were incubated in 2 µg/ml anti-CD3/28-coated culture plates with 100 U/ml rIL-2 for 48 h and cultured for an additional 3 days with 100 U/ml rIL-2. The purity of each population was confirmed to be more than 95% by flow cytometry.

### Animals

IGSF4 transgenic mice (B6.Cg-Tg(CAG-Cadm1)) were obtained from RIKEN BioResource Research center. C57BL/6 wild-type mice and *OTI* and *OTII* TCR transgenic mice (C57BL/6 background) were purchased from Jackson Laboratories (Bar Harbor, ME, USA). IGSF4 Tg mice were crossed with *OTII* mice to generate an OVA-specific TCR transgenic line. All mouse lines were confirmed by PCR using genomic DNA. All mice were housed in specific pathogen-free conditions, and all experiments were approved by the Animal Care and Use Committee of the School of Life Sciences, Gwangju Institute of Science and Technology.

### Plasmid Constructs

All of the IGSF4-deletion, chimeric, or swapping mutants and CD3 ϛ chimeras were generated by overlapping PCR, and the products were incorporated into pEGFP-N1, dsRed_N1 (CMV promoter; Takara Bio Inc.), pCS4-Myc (Addgene), or modified pHJ1 lentiviral vector. Targeted amino acid mutations and TM-domain swapping are described in **Figure 2**. For retroviral transduction to mouse primary T cells, wt-IG4 and IG4ΔEXT genes were subcloned into the modified pRV-IRES-GFP vector. All chimeric mutants were confirmed by sequencing DNA in expression vectors.

### RT-PCR and Real-Time Quantitative PCR

Total RNA was isolated from cells with TRI reagent (Molecular Research Center, Cincinnati, OH, USA) and reverse transcribed using RT-premix (Intron Biotechnology).

PCR was performed with the following primers (the respective forward and reverse pairs are indicated): human IL-2, 5’-CACGTCTTGCACTTGTCAC-3’ and 5’-CTTCTTGGGC- ATGTAAAACT-3’; human GAPDH, 5’-CGGAGTCAACGGATTTGGTCGTAT-3’ and 5’-AGCCTTCTCCATGGTGGTGAAGAC-3’; human IGSF4, 5’-AAGTAGTCCTGAAG GACAGAAACT-3’ and 5’- ATAAATCAGCATAAGTTTTCCACA-3’. The expression levels of *IGSF4* and *hIL*-2 were evaluated by quantitative PCR. Amplification was performed in a StepOne Real-Time PCR System (Applied Biosystems; Norwalk, CT, USA) for continuous fluorescence detection in a total volume of 10 μl of cDNA/control and gene-specific primers using SYBR Premix Ex *Taq* (Takara Bio). The mRNA levels of the target genes were normalized relative to those of *Gapdh* using the following formula: relative mRNA expression = 2^−(ΔCt of target gene − ΔCt of GAPDH)^, where Ct is the threshold cycle value. In each sample, the expression of the analyzed gene was normalized to that of *Gapdh* and described as the mRNA level relative to *Gapdh*.

### Cell Transfection and Viral Infection

Transient transfection to MT4 cells was performed by Amaxa technology using the human T-cell line nucleofector kit V (Lonza). For *IGSF4* knockdown, 10 nM siRNAs were introduced into target cells and cultured for 48 h before use. Transfection to HEK293T cells for construct expression was performed using Lipofectamine 2000 (Life Technologies). To establish stable cell lines, cDNA in pHJ-1 lentiviral vector was co-transfected with lentiviral packaging vectors (pHDM-Hgpm2, pRC/CMV-Rev1b, and pHDM.G) into HEK293T cells. The supernatants were then collected and spin-infected into Jurkat T cells by centrifugation at 2,000 × g at 25°C in the presence of 8 μg/ml polybrene (Sigma-Aldrich). For retroviral infection, a total of 1 × 10^6^ retroviral packaging cells (Plat-E; Cell Biolabs) were seeded overnight in 6 cm^2^ dishes. Retroviral particles were generated by transfection with retroviral vectors (EV, wt-IG4, and IG4Δ;EXT) and pCL-Eco packaging vector using Lipofectamine 2000 (Invitrogen). After 48 h, virus supernatants (2.5 ml) were harvested, mixed with 1×10^6^ mouse T cells in 12-well plates coated with 20 μg/ml retronectin (Clontech, Mountain View, CA, USA), and centrifuged at 22,000 × g at 25°C for 90 min with rIL-2 (100 U/ml). The transduced T cells were maintained with fresh media with rIL-2 and expanded for 3 days. The percentage of cells expressing GFP was measured at 48 h post-infection. For retroviral transduction, mouse CD8^+^ T cells were incubated in 2 μg/ml anti-CD3/28-coated plates with rIL-2 (100 U/ml) for 48 h.

### T-Cell Stimulation

Jurkat T cells and mouse T cells were stimulated with either plate-bound anti-CD3 (10 µg/ml OKT3 for human; 10 µg/ml 145-2C11 for mouse) or 2 µg/ml CD28. For superantigen stimulation, Jurkat T cells were incubated with 1 µg/ml SEE–pulsed Raji B cells for the indicated time. CD4^+^ T cells from *OTII*-crossed mice were incubated with 1 µg/ml OVA-pulsed CD11C^+^ DCs. CD8^+^ T cells expressing RV, wt-IG4, or IG4ΔEXT were incubated with B16F10 in the presence or absence of OVA peptide (257-264, 1 μg/ml) at 37°C for 30 min.

### ELISA

Jurakt T cells, CD4^+^ T cells, or CD8^+^ T cells (1 × 10^6^ cells/sample) were stimulated as described in the T-cell stimulation section. After 24-48 h, the amounts of IL-2 and IFN-γ in the supernatants from three replicas for each condition were determined by ELISA with Duo Set Mouse ELISA kits for IL-2 and IFN-γ (R&D Systems).

### Immunoprecipitation

For immunoprecipitation, HEK293T cells were harvested at 48 h post-transfection, washed quickly once in cold PBS, and lysed in 1% Triton X-100 lysis buffer containing 20 mM Tris-HCl, pH 7.4, 150 mM NaCl, one tablet of Complete protease inhibitors (Roche), and phosphatase inhibitors (cocktails I and II; Sigma-Aldrich). The lysates of equivalent protein content were precleared on Sepharose 4B (GE Healthcare) for 1 h at 4°C. GFP-fused IGSF4 and mutant proteins were immunoprecipitated with anti-GFP-conjugated Sepharose 4B. Immunoprecipitates were washed twice with the corresponding 1% Triton X-100 lysis buffer and twice with a lysis buffer without detergent. The proteins were resolved by 10–12% SDS-PAGE, and then western blot analysis was performed.

### Western Blotting

The cells were lysed in ice-cold lysis buffer (50 mM Tris-HCl, pH 7.4, containing 150 mM NaCl, 1% Triton X-100, and one tablet of complete protease inhibitors) for 15 min on ice. Cell lysates were centrifuged at 16,000 × g for 30 min at 4°C, and the supernatants were eluted with sodium dodecyl sulfate (SDS) sample buffer (100 mM Tris-HCl, pH 6.8, 4% SDS, and 20% glycerol with bromophenol blue) and heated for 5 min. The proteins were separated by SDS polyacrylamide gel electrophoresis (PAGE) on 10–15% gels and were transferred to nitrocellulose membranes using a Trans-Blot SD semidry transfer cell (Bio-Rad, Hercules, CA). The membrane was blocked in 5% skim milk (1 h), rinsed, and incubated with the appropriate antibodies in TBS containing 0.1% Tween 20 (TBS-T) and 0.5% skim milk overnight. Excess primary antibody was then removed by washing the membrane three times in TBST. The membrane was then incubated with 0.1 μg/ml peroxidase-conjugated secondary antibodies (anti-rabbit or anti-mouse) for 1 h. After three washes with TBST, bands were visualized using western-blotting detection reagents and were then exposed to X-ray film (Kodak, Rochester, NY).

### Confocal Microscopy

For translocation analysis, Jurkat T cells expressing wt-IG4_GFP or other mutants were incubated with SEE-pulsed Raji B cells stained with CellTracker orange CMRA (Invitrogen) for 30 min and placed on PLL-coated coverslips or anti-CD3-antibody-coated coverslip for 30 min. The accumulation of wt-IG4_GFP or other mutants at the T cell-APC contact site or anti-CD3-coated surface was calculated as the ratio of fluorescence intensity at the contact region (Fcon = c) to the fluorescence intensity at the opposite site (Foppo = o). Fluorescence intensity was represented by an intensity profile, with blue indicating the lowest intensity and red indicating the highest intensity. To evaluate CD3 ϛ−clustering in MT4 cells, scrambled or IGSF4 siRNA-treated cells were transfected with CD3 ϛ_GFP using Amaxa. The cells were then placed on PLL at 24 h post-transfection and imaged using a 100×, NA 1.40 oil immersion objective lens on a laser-scanning confocal microscope (FV1000; Olympus, Tokyo, Japan). CD3 ϛ_GFP clustering was identified using the “find objects using intensity” and “separate touching objects (object size guide 0.2 μm^2^) functions of Volocity imaging analysis software (PerkinElmer).

### Total Internal Reflection Fluorescence Microscopy

To measure the number and area of TCR microclusters, EV- or IG4ΔEXT-*OTI* CD8^+^ T cells were stained with TCRβ (H57Fab-594). The cells were then placed on a planar lipid bilayer with OVA257-265-H-2K^b^/ICAM-1 and immediately imaged for 20 min by TIRFM (IX-81; Olympus, Tokyo, Japan) equipped with a solid-state laser (488 nm, 20 mW; Coherent, Santa Clara, CA, USA). The generation of bilayers has been described previously (29, 30). Microclusters were identified using the “find objects using intensity” and “separate touching objects (object size guide 0.2 μm^2^) functions of Volocity imaging analysis software (PerkinElmer). In this analysis of lipid bilayer imaging, only the clusters generated within 1 min after attachment were analyzed.

### Flow Cytometric Analysis

The retroviral transduction efficiency and surface expression of CD69 were analyzed by flow cytometry. CD8^+^ T cells (1 × 10^6^ cells/sample) were stimulated as described in the T-cell stimulation section, suspended in PBS containing 2% FBS, stained with APC-conjugated CD69 for 10 min at RT, and washed with PBS. Cells were then assessed on a FACS Canto instrument (BD Biosciences), and the data were analyzed with FlowJo software (TreeStar).

### Tetramer Staining for Avidity Measurement

EV-, wt-IG4-, or IG4ΔEXT-*OTI* CD8^+^ T cells were washed twice with PBS and stained with 1 µg/ml of APC-conjugated OVA-H-2K^b^ tetramers per 1 × 10^6^ cells at 4°C for 20 min. As a negative control, 1 µg/ml of APC-conjugated TRP2-H-2K^b^ was used. The cells were washed again with PBS and analyzed *via* flow cytometry. Tetramer intensity plots were obtained from the gated on EV- or IG4ΔEXT-*OTI* CD8^+^ T cells that highly express GFP.

To measure the dissociation rates of OVA-H-2K^b^ tetramers, transduced cells were incubated with 1 µg/ml of APC-conjugated OVA-H-2K^b^ tetramers for 1 h at 22°C. Cells were pelleted and resuspended in 200 μl FACS buffer in the presence of 100 μg/ml anti-mouse MHC class I (H-2K^b^) antibody on ice. The fluorescence intensities of 10,000 cells were measured at different time points after adding the anti-mouse MHC class I (H-2K^b^) blocking antibody. The geometric mean fluorescence (GMF) of tetramer at time 0 was presented as 100% and the % of maximum binding that associated with the cells after various times was shown. Non-linear regression curve was fitted into normalized data and R squares and t_1/2_ were determined using the Prism software.

### Proliferation Assay

EV-, wt-IG4-, or IG4ΔEXT-*OTI* CD8^+^ T cells were stained with CellTrace™ Violet (CTV) and co-cultured with 1 μg/ml of pOVA (257–265)-pulsed DCs for 24 h. Total cells were fixed with Cell Fixation and Cell Permeabilization Kit (Thermo Fisher Scientific) for 1 h at 4°C and stained with anti-Ki67 along with 1 μl of NucSpot Far-Red for 1 h at 4°C in the dark. The samples were washed with PBS and analyzed by flow cytometry. The percent of proliferative populations was acquired from the gate in a CTV-positive population.

### Phenotyping of CD8^+^ Cells

Homogenized splenocytes from *OTI* mice were stimulated with 1 μg/ml of pOVA (257-265) (day 0). Cells were harvested at day 2 and CD8^+^ T cells were purified using an EasySep magnetic separation system and transduced with retroviral particles carrying EV-, wt-IG4-, or IG4ΔEXT. Infected cells were further cultured and harvested on day 3, 7, 14, and 21 post-infection to assess their memory phenotype. Cells were stained with APC-labeled anti-CD62L and Percp-Cy5.5-labeled CD44.

### *In Vitro* Cytotoxicity Assay

B16F10 cells were stained with cell tracker orange CMRA (Invitrogen) and viral-transduced *OTI* CD8^+^ T cells were incubated with cancer cells in the presence or absence of OVA peptide (257–264, 1 μg/ml) at a 5:1 ratio in 5 ml round-bottom polystyrene tubes at 37°C for 4 h. After incubation, 10 μL of a 5 μg/ml solution of 7-AAD was added to the cell suspension for 10 min at RT. The cells were evaluated on a FACS Canto (BD Biosciences), and the data were analyzed with FlowJo software (TreeStar).

### Tumor Models

For the solid or metastasis tumor model, B16F10-OVA cells (3 ×10^5^) were inoculated at the dorsal flank region of C57BL/6-recipient mice (8 weeks old) subcutaneously and intravenously, respectively. *OTI* CD8^+^ T cells carrying EV, wt-IG4, or IG4Δ;EXT (1 × 10^7^) were injected *via* IV route on days 7, 10, and 13 after tumor inoculation. Mice were sacrificed at day 21 post-inoculation of tumor cells. At the end of the experiments, tumors were isolated, weighed, and photographed for gross images. Tumor volume was measured using calipers every 3 days from the time T cells were injected and was calculated based on the formula: length × width × height (mm^3^).

### Statistical Analysis

Mean values were calculated using the data obtained from at least three independent experiments conducted on separated days. Unpaired Student’s t tests and one-way analyses of variance (corrected for all pairwise comparisons) were performed using Prism software. Differences between groups were considered significant when the *P* < 0.05.

## Results

### IGSF4 Is Involved in TCR-Mediated Signaling and T Cell Activation

**Figure 1:**
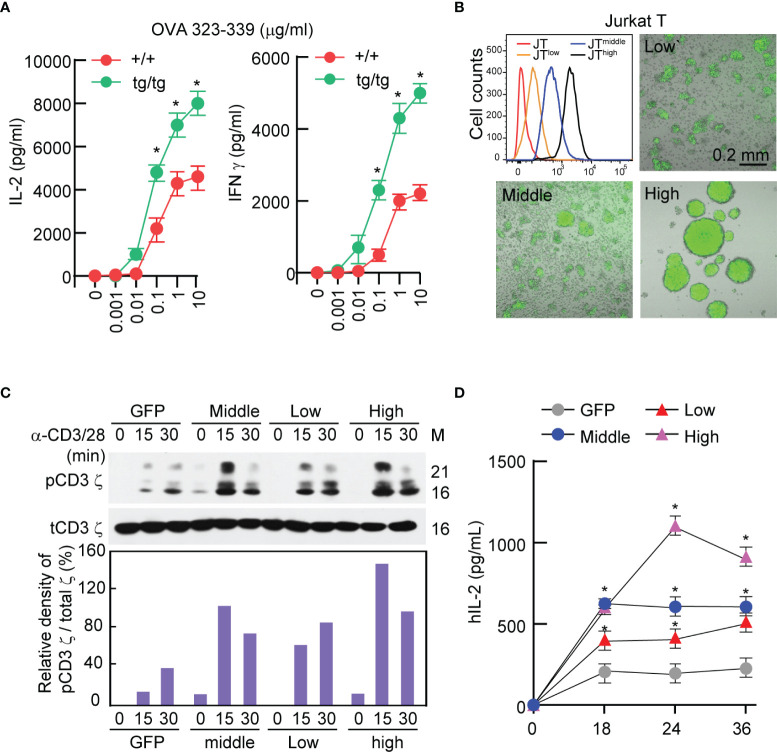
IGSF4 is involved in T cell antigen receptors (TCR)-mediated signaling and T-cell activation. **(A)** Naïve *OTII* TCR CD4^+^ T cells from wild-type or IGSF4 Tg mice were stimulated with the indicated concentrations of OVA peptide 323-339. IL-2 and IFN-γ secretion levels in the supernatant were evaluated by ELSA at 24 h after stimulation. Data represent the means of three experiments ± SEM. *P < 0.001 *vs.* wild-type (+/+) **(B)** Jurkat T cells expressing IGSF4_GFP were sorted into JT^low^, JT^middle^, and JT^high^ by flow cytometry based on GFP intensity. Fluorescence and DIC images of each cell line were acquired by confocal microscopy. **(C)** Each cell line from B was stimulated with anti-CD3/28 for the indicated time points and then analyzed by western blotting with specific antibodies for pCD3 ϛ or t-CD3 ϛ (top). Densitometric analysis was performed with ImageJ software and used to determine the relative intensities of bands corresponding to pCD3 ϛ, which were normalized to those of t-CD3 ϛ (bottom). **(D)** IL-2 secretion levels in the supernatant were evaluated after stimulation of each cell line with anti-CD3/28 at the indicated time points. Data represent the means of three experiments ± SEM. *P < 0.001 *vs.* GFP.

It has been previously reported that IGSF4-deficient mice exhibit defective T-cell function (28). In contrast, *OTII TCR* T cells isolated from IGSF4-transgenic mice exhibited similar levels of cytokine production as wild-type *OTII TCR* T cells at an approximately 10-fold lower antigen density (**Figure 1A**), suggesting that IGSF4 is involved in the amplification of TCR signaling. To test this hypothesis, Jurkat T cells expressing low, middle, and high levels of IGSF4_GFP (IG4G) were generated, and experiments were conducted to test whether the expression levels of IGSF4 influence the magnitude of TCR signaling (**Figure 1B**). Overexpression of IGSF4_GFP proportionally increased the homotypic aggregation of Jurkat T cells due to homotypic *trans*-interaction between IGSF4 molecules (**Figure 1B**) (31). Interestingly, the levels of phospho-CD3 ζ-chain were strongly correlated with the levels of IGSF4 expression (**Figure 1C**). Moreover, the amounts of hIL-2 released from the Jurkat T cells were correlated with the expression levels of IGSF4 (**Figure 1D**). Collectively, these findings strongly evidence that IGSF4 is directly involved in TCR-mediated signaling and hence T-cell activation.

### The TM Domain of IGSF4 Mediates Binding to the CD3 ζ-Chain and Enhances TCR Signaling

**Figure 2:**
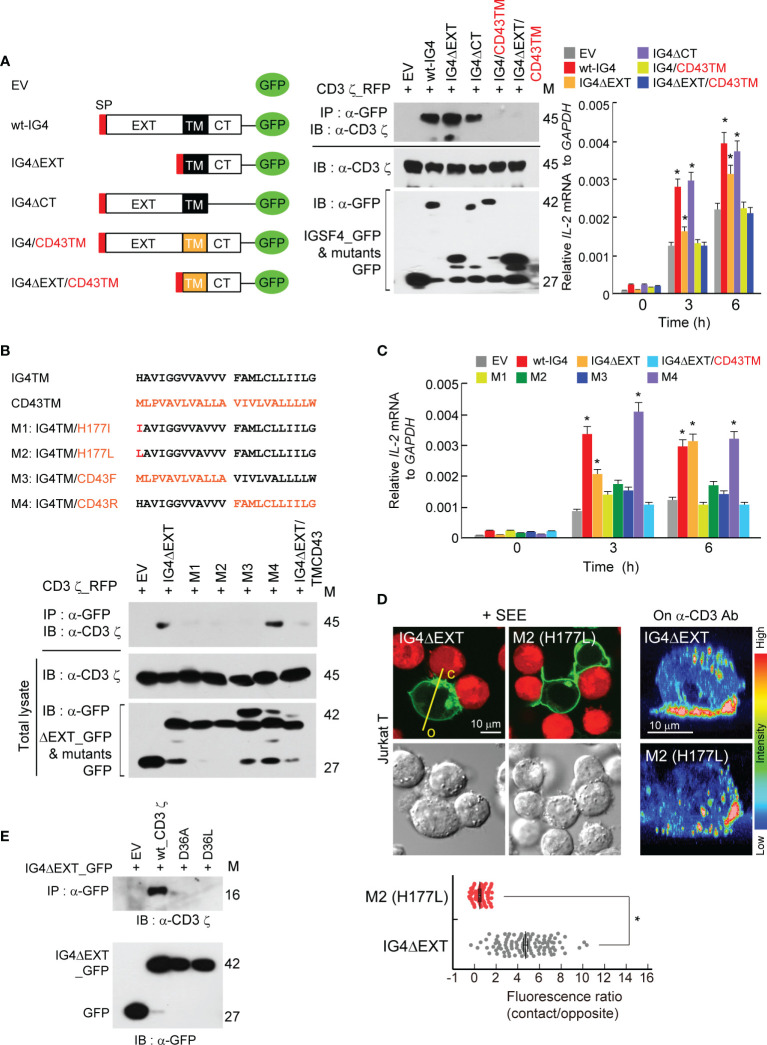
Transmembrane (TM) domain of IGSF4 mediates binding to the CD3 ζ-chain and enhances T cell antigen receptors (TCR) signaling. **(A)** Schematic diagram showing deletion and swapping mutants of IGSF4 constructs (left). Black and orange colors of TM stand for the IGSF4 (IG4) and CD43 TM regions, respectively. Immunoprecipitation and immunoblotting of EV, wt-IG4, or the indicated mutants with CD3 ϛ co-expressed in HEK293T cells (middle). Jurkat T cells overexpressing indicated constructs were stimulated with anti-CD3/28, and *IL-2* mRNA levels were assessed by real-time quantitative PCR (right). Data represent the means of three experiments ± SEM. *P < 0.001 *vs.* EV. **(B)** Amino acid sequences of the IGSF4 TM, the CD43 TM, and their mutants (M1-M4). Residues in red of the mutants indicate amino acid substitutions (top). Immunoprecipitation and immunoblotting of indicated constructs with CD3 ϛ co-expressed in HEK293T cells. **(C)** Jurkat T cells overexpressing the indicated constructs in **(B)** were stimulated with anti-CD3/28, and *IL-2* mRNA levels were assessed by real-time quantitative PCR (graphs). Data represent the means of three experiments ± SEM. *P < 0.001 *vs.* EV **(D)** Jurkat T cells expressing IG4ΔEXT or M2 mutant were either incubated with SEE-loaded Raji B cells (red) or placed on coverslips coated with anti-CD3, and confocal analysis was performed. The images on anti-CD3 were reconstituted to three-dimensional images by the FLUOVIEW program. Note, c = contact region and o = opposite region. Each dot represents a single measurement, and at least 50 cells were examined. Data represent the means of three experiments ± SEM. *P < 0.001 *vs.* IG4ΔEXT **(E)** Immunoprecipitation and immunoblotting of indicated CD3 ϛ mutants (D36A or D36L) with IG4ΔEXT. The data in **(A, B, E)** are representative of at least three independent experiments.

It is understood that IGSF4 enhances TCR signaling, but how IGSF4 upregulates TCR signaling is still unknown. As we previously observed that IGSF4 directly binds to the CD3 ζ-chain through the TM domain (28), we further investigated whether IGSF4 action is associated with TM–TM interaction between IGSF4 and the CD3 ζ-chain. To this end, we primarily corroborated previous results using mutants of IGSF4 (28). In addition, to examine their direct interaction, HEK293 T cells were utilized to co-express both proteins. Among various mutants, the constructs that contain the TM domain of IGSF4 did not only bind to the CD3 ζ-chain in HEK293 T cells, but also had an increased *hIL2* mRNA expression in Jurkat T cells (**Figure 2A**). Overexpression of the deletion mutant of the extracellular domain of IGSF4 (IGΔEXT) significantly increased *hIL2* mRNA expression, while the level was lower than that of wild-type IGSF4 (wt-IG4) or the deletion mutant of the cytoplasmic domain (IGΔCT) (28) (**Figure 2A**).

Next, the study aimed to determine which amino acid(s) in the TM region is/are critical in mediating the interaction between IGSF4 and the CD3 ζ-chain. The TCR-CD3 complex is primarily assembled by the basic and acidic residues located in the TM domains of TCR and CD3 molecules, respectively (32). In addition, it has been proposed that the CD3 ζ-chain interacts with the TCR chain through Asp36 (32). Since the imidazole side chain of histidine is partially protonated, we considered whether this residue can interact with the aspartic acid (Asp36) of the CD3 ζ-chain TM domain. Among four mutants tested, mutants with His177Ile (M1), His177Leu (M2), or an exchange of the front IGSF4TM with CD43TM (M3) failed to bind to the CD3 ζ-chain (**Figure 2B**), demonstrating that His177 residue is critical for binding to the CD3 ζ-chain. In addition, three mutants (M1, M2, and M3) showed significant reductions in *hIL2* mRNA expression (**Figure 2C**). Moreover, M1 and M2 mutants lost their capacity to accumulate at the immunological synapse (IS) (**Figure 2D**). Further, we exchanged the Asp36 of the CD3 ζ-chain with Ala or Leu to make D36A or D36L. Strikingly, the mutation at Asp36 caused a complete loss of the interaction between IGSF4 and the CD3 ζ-chain (**Figure 2E**), demonstrating that the interaction between His177 of the IGSF4 TM and Asp36 of the CD3 ζ-chain TM is critical in forming IGSF4—CD3 ζ dimers.

### IGSF4 Is *cis*-Dimerized Through a TM Domain That Increases TCR Clusters

**Figure 3:**
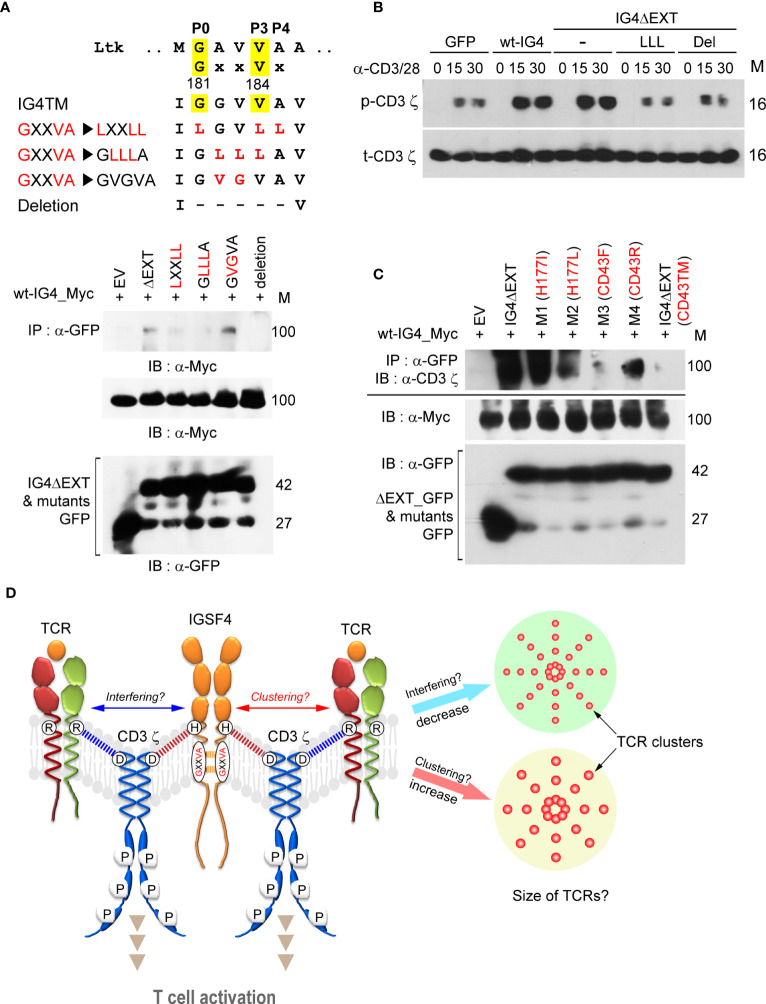
IGSF4 is *cis*-dimerized through the transmembrane (TM) domain, which increases T cell antigen receptors (TCR) clusters. **(A)**. IGSF4 TM (IG4TM) sequences showing the GxxVx motif and mutated (highlighted in red) or deleted regions (top). Immunoprecipitation and immunoblotting of indicated constructs with wt-IG4_Myc co-expressed in HEK293T cells. **(B)** Cells from **(A)** were stimulated with anti-CD3/28 for the indicated time points and then analyzed by western blotting with specific antibodies for p-CD3 ϛ or t-CD3 ϛ. **(C)** Immunoprecipitation of the indicated constructs with wt-IG4_Myc co-expressed in HEK293T cells. Immunoblotting was performed using antibodies against the CD3 ϛ-chain. The data in **(A–C)** are representative of at least three independent experiments. **(D)** Illustration of the proposed model. The results suggest that IGSF4 is *cis*-dimerized *via* the GxxVx motif in the TM domain and also interacts with the CD3 ϛ-chain *via* histidine through charge-interaction. Overexpression of IGSF4 may increase the TCR clustering that enhances TCR avidity. Otherwise, IGSF4 might interfere with TCR cluster formation as it contains a binding residue (Asp36) with CD3 ζ and TCRαβ chains.

To further understand the molecular details of the IGSF4-CD3 ζ assembly, we determined whether the IGSF4 TM domain also contains a homo-dimeric motif. Indeed, it is well-understood that the CD3 ζ-chain possesses a dimeric motif (LxxxxGVxxT) in the TM, thereby mediating the TCR/CD3 complex assembly with CD3γδ TM domains (33). It has been known that IGSF4 forms a dimer through the second Ig-like domain of the extracellular region (31). However, dimerization through the TM region may better facilitate IGSF4—CD3 ζ complex assembly. Interestingly, the IGSF4 TM contains a GxxVx sequence, which is similar to the dimerization motifs in the TM domain of tyrosine kinase growth factor receptors such as Ltk (mouse leukocyte tyrosine kinase; GxxVx) (34). Therefore, this site was exchanged with the amino acid Leu to generate LxxLL or GLLLA. Two mutants failed to be dimerized, as determined by immunoprecipitation with wt-IG4 (**Figure 3A**). Deletion of the GxxVx motif also caused the loss of the dimeric potential of the IGSF4 TM domain (**Figure 3A**). In contrast, the mutation from G**GV**VA to G**VG**VA did not alter the TM–TM interaction (**Figure 3A**), suggesting that Gly181 and Val184 are important amino acids for the IGSF4 TM–TM interaction. As shown in **Figure 3B**, LLL and deletion mutants showed reduced CD3 ζ-phosphorylation in response to TCR activation. This study also tested whether the His177 mutation may hinder the homo-dimeric TM–TM interaction; however, the His177 mutation did not affect TM–TM interactions (**Figure 3D**). Loss of the TM–TM interactions in CD43F (M3) or CD43TM mutants further suggest that the GxxVx motif is critical for the self-dimerization of the TM domain (**Figure 3D**).

From the results thus far, we hypothesized that IGSF4 can induce multimeric TCR clusters, not only by forming a homodimer through its TM domain but also by interacting with CD3 ζ. On the contrary, however, IGSF4 might interfere with TCR cluster formation since it shares a binding residue (Asp36) with CD3 ζ for TCRαβ chains. **Figure 3E** represents the summarized schematic diagram of the IGSF4 interaction with the CD3 ζ-chain, which may lead to the clustering or interference of multimeric TCRs.

**Figure 4:**
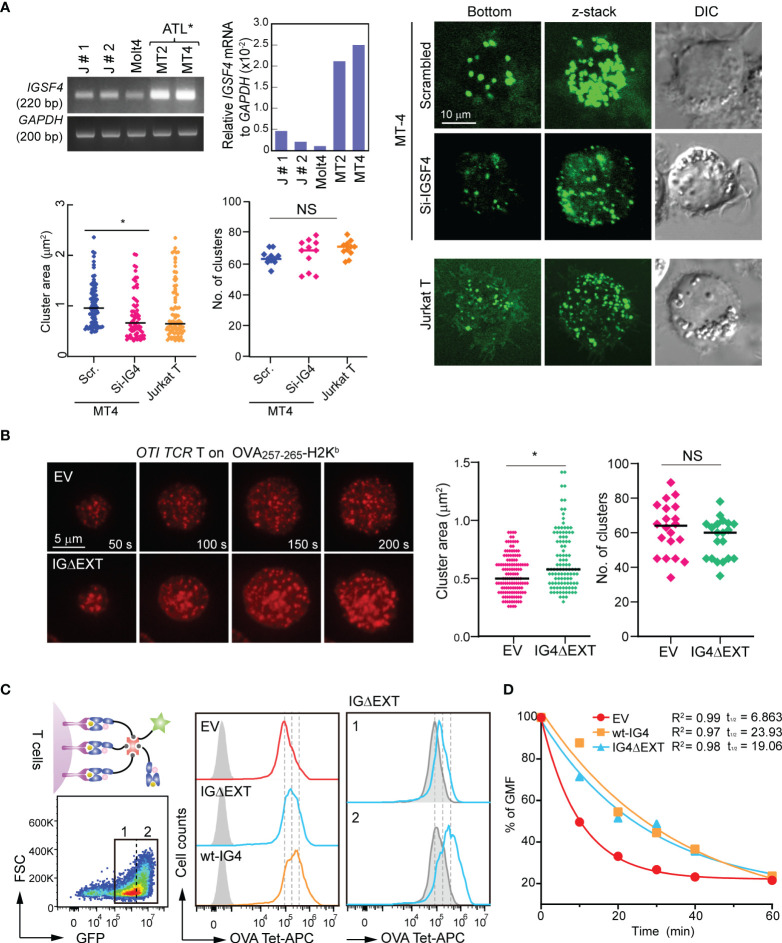
IGSF4 transmembrane (TM) domain increases T cell antigen receptors (TCR) pre-clusters and enhances TCR avidity in T cells. **(A)** Conventional and real-time PCR measurements of *IGSF4* mRNA expression in various cell lines (top). Exogenous CD3 ϛ_GFP clustering was analyzed in MT4 T cells transfected with scrambled- or siRNA-targeting IGSF4 by confocal microscopy (bottom). Jurkat T cells were used as a control to compare the sizes of TCR pre-clusters. Average areas and the number of exogenous CD3 ϛ_GFP clustering were analyzed by Volocity software. *P < 0.001 *vs.* si-scrambled. **(B)** *OTI* CD8^+^ T cells expressing EV or IG4ΔEXT were stained with anti-TCRβ (H57Fab-Alexa594) and examined on a planar bilayer presenting OVA257-265-H-2K^b^/ICAM-1. Snapshots of every 50 sec of the time lapse are shown. Average areas and the number of TCR microclusters observed after initial cell/bilayer contact within 1 min were analyzed using Volocity software. **(C)** *OTI TCR* T cells expressing EV, wt-IG4, or IG4Δ;EXT (left) were gated based on GFP-positive populations (1 + 2, left) and analyzed binding of OVA-tetramer-APC (middle). IG4Δ;EXT cells were further gated based on the expression levels of IGSF4 (1= low and 2= high), and the binding intensity of OVA-tetramer-APC was analyzed (right). **(D)** Dissociation kinetics of OVA-tetramer-APC binding to *OTI TCR* T cells expressing EV, wt-IG4, or IG4ΔEXT at 22°C. The half-lives (*t*_1/2_) of a tetramer were determined using real-time flow cytometry in the presence of saturating amounts of anti-H-2K^b^ blocking antibody.

Next, we examined whether IGSF4 affects the size of TCR clusters. To this end, MT2 and MT4 cells were selected, as these cells express high levels of IGSF4 (**Figure 4A**) but low levels of TCR and CD45 (35–37). Interestingly, overexpression of the CD3 ζ-chain dramatically increased puncta-like CD3 ζ clusters, while siRNA targeting of IGSF4 decreased them (**Figure 4A**). We further tested the effect of the IGSF4 TM domain in mouse *OTI TCR* CD8^+^ T cells. In contrast to the EV (GFP), *OTI* T cells expressing IG4ΔEXT showed an increase in TCR clusters, while the numbers of TCR clusters did not change (**Figure 4B**). To further confirm the avidity increase by IGSF4, we measured the TCR-pMHC binding strength using OVA-tetramer (H-2K^b^) conjugated with APC. *OTI* T cells expressing wt-IGSF4 (*OTI* wt-IG4-CD8^+^ T) and IG4ΔEXT (*OTI* IG4ΔEXT-CD8^+^ T) showed a significant increase in OVA-tetramer binding but not negative control TRP2-tetramer binding, as compared to *OTI* T cells expressing EV (*OTI* EV-CD8^+^ T cells (**Figure 4C**). In addition, the dissociation rate indicated that *OTI* wt-IG4-CD8^+^ T and *OTI* IG4ΔEXT-CD8^+^ T cells had an increased half-life of OVA-tetramer binding compared to *OTI* EV-CD8^+^ T cells (**Figure 4D**).

### Overexpression of IG4ΔEXT Enhances the Antitumor Activity of CD8^+^ T Cells *In Vitro* and *In Vivo*

The ability of IGSF4 to influence the sizes of TCR clusters *via* the TM domain suggests that this protein may be an attractive candidate for CD8^+^ T-cell-mediated cancer immunotherapy. This is because, until now, it has been thought that the avidity of TCR would play an important role in the antitumor responses of TILs or engineered-TCR T cells, but there have not been any reports, to our knowledge, that have evaluated the antitumor response by directly controlling the cluster sizes of TCRs. Therefore, this study investigated whether the TM domain of IGSF4 could affect the antitumor activity of CD8^+^ T cells by enhancing TCR signals.

**Figure 5:**
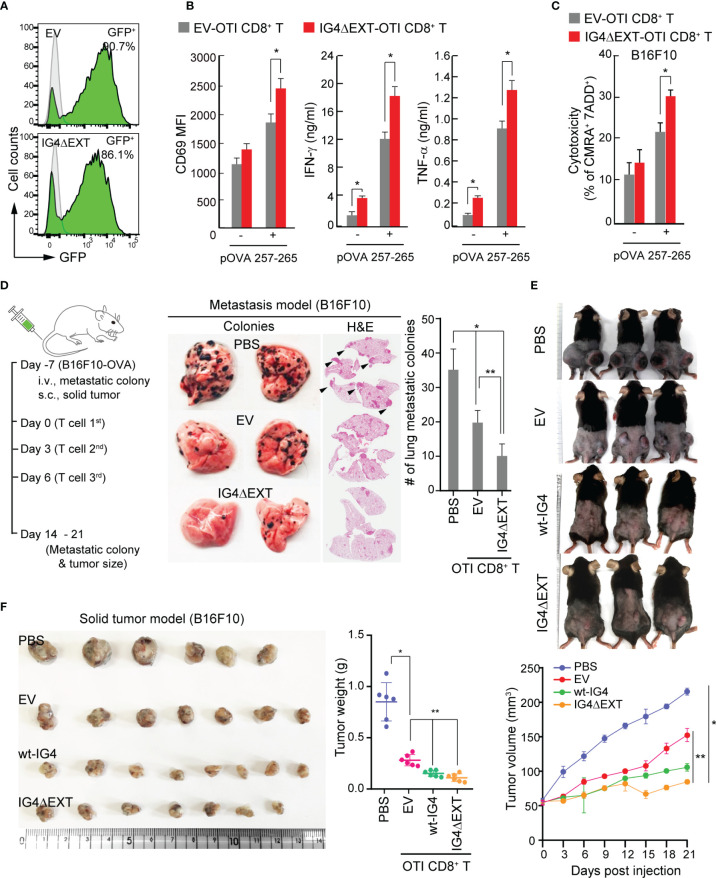
Overexpression of IG4ΔEXT enhances the antitumor activity of CD8^+^ T cells *in vitro* and *in vivo*. **(A)** Efficiency of the retroviral transduction of EV or IG4ΔEXT in CD8^+^ T cells from *OTI* mouse. **(B)** EV- or IG4ΔEXT-*OTI* CD8^+^ T cells were incubated with B16F10 in the presence or absence of OVA 257-265 for 9 h, and then the surface expression of CD69 was measured. IFN-γ and TNF-α were measured at 24 h post-incubation with B16F10 in the presence or absence of OVA peptide. *P < 0.001 *vs.* EV-*OTI* CD8^+^ T cells. **(C)** Target cells from **(B)** were stained with 7-AAD, and dead cells were counted by flow cytometry. **(B, C)** Data represent the means of three independent experiments. *P < 0.001 *vs.* EV-*OTI* CD8^+^ T cells **(D)** Schematic diagram of the metastasis and solid tumor mouse models (left). Gross and H&E staining images of B16F10 lung metastatic nodules from C57BL/6 mice administered *via* IV injection with PBS, EV-, or IG4ΔEXT-*OTI* CD8^+^ T cells (right). Arrowheads indicate metastatic foci. The metastatic colonies were quantified and represented as a bar graph. Data represent the means of three independent experiments (n=4 mice per group). *P < 0.005 *vs.* PBS, **P < 0.001 *vs.* EV-*OTI* CD8^+^ T cells. **(E)** Images of B16F10 solid tumors obtained from mice. Tumor volumes were measured using calipers every 3 days after PBS or T cell (EV*-OTI* CD8^+^ T, wt-IG4- *OTI* CD8^+^ T, and IG4ΔEXT-*OTI* CD8^+^ T) injection until day 21. **(F)** In some cases, tumors were harvested at day 14 from the mice described above **(E)**. Tumor weights were measured at the end of the experiments. Data are representative of four independent experiments (n=6 mice per group for each experiment). *P < 0.005 *vs.* PBS, **P < 0.001 *vs.* EV-*OTI* CD8^+^ T cells.

Retroviral DNA constructs containing GFP (empty vector [EV]) or IG4ΔEXT_GFP were generated. Both GFP and IG4ΔEXT_GFP were highly expressed in mouse *OTI* TCR CD8^+^ T cells (**Figure 5A**). The cytokines IFN-γ and TNF-α and the expression of CD69 were evaluated after *OTI* TCR CD8^+^ T cells were incubated with OVA^+^-B16F10 melanoma cells. *OTI* TCR CD8^+^ T cells expressing IG4ΔEXT_GFP (*OTI* IG4ΔEXT-CD8^+^ T) significantly upregulated CD69 expression and cytokine release (**Figure 5B**). Moreover, cytotoxic activity was also enhanced in *OTI* TCR CD8^+^ T cells expressing IG4ΔEXT_GFP (**Figure 5C**), suggesting that TCR avidity positively regulates the antitumor response *in vitro*.

Since the *in vitro* results revealed that *OTI* IG4ΔEXT-CD8^+^ T cells showed cytokine release and higher cytotoxicity, the effectiveness of *OTI* IG4ΔEXT-CD8^+^ T cells was then examined *in vivo*. To this end, OVA^+^-B16F10 melanoma cells were used for two independent models. The metastasis and solid tumor models are schematically represented in **Figure 5D**. After 7 days of intravenous (IV) injection with OVA^+^-B16F10 cells, *OTI* IG4ΔEXT-CD8^+^ T cells were adoptively transferred *via* IV injection three times. The metastatic colonies were evaluated at 14 days from the first injection, and a significant reduction in metastatic colonies was observed in mice with adoptively transferred *OTI* IG4ΔEXT-CD8^+^ T cells (**Figure 5D**). For the solid tumor model, OVA^+^-B16F10 cells were implanted into the mammary fat pads of C57BL/6 female mice. Seven days later, the mice were randomized into four groups for administration of PBS alone, *OTI* EV-CD8^+^ T, *OTI* wt-IG4-CD8^+^ T, and *OTI* IG4ΔEXT-CD8^+^ T cells (**Figure 5D**). Tumor volumes were measured every 3 days after T cell injection until day 21 (**Figure 5E**). In some cases, animals were sacrificed 14 days after tumor injection, and the tumors were weighed (**Figure 5F**). The average volume and weight of the tumors from mice injected with *OTI* IG4ΔEXT-CD8^+^ T and *OTI* wt-IG4-CD8^+^ T cells were lower than those of the tumors from mice injected with *OTI* EV-CD8^+^ T cells (Figs. 5E, F). Collectively, the present study provides the first evidence that the direct control of TCR avidity can potentiate the antitumor response of CD8^+^ T cells *in vivo*.

## Discussion

Cytotoxic T cells are a highly attractive subset of immune cells for cancer immunotherapy, as they have the capacity to exhibit remarkable antigenic specificity through T cell antigen receptors (TCR). However, engineered high-affinity TCR-targeting tumor-associated antigens have faced the paradigm of central tolerance due to induced autoimmune responses (6). Thus, some reports have suggested that TCR avidity control is another option for potentiating the capacity of TCR signaling (22, 38, 39). However, no direct evidence has been reported yet regarding the direct control of TCR avidity in the antitumor response of cytotoxic T cells. The present study found that the TM domain of IGSF4 can increase the sizes of TCR clusters through its molecular abilities to bind with the CD3 ζ-chain and to self-assemble, the latter of which enhances the avidity of TCR without any influence on affinity. Interestingly, expression of the TM domain (without the ectodomain) alone could potentiate the antitumor response of cytotoxic T cells, thereby evidencing that avidity control is an important strategy for enhancing T-cell potency.

IGSF4 is expressed in most tissues, but at very low levels in peripheral blood lymphocytes, probably due to hyper-methylation at the promoter region (40). However, the positive correlation between the levels of IGSF4 and the expression of IL-2 denotes a function of IGSF4 in T-cell activation. In this regard, the low expression of IGSF4 may have a significant meaning with respect to T-cell immunity; if IGSF4 is highly expressed in T cells, the property of IGSF4 to form a homodimer may result not only in homotypic clumping of the T cells themselves, but also in adhesion to the IGSF4-positive cells or tissues in the body, thereby inducing self-damage. Therefore, the tight regulation of IGSF4 expression in T cells would be important for T-cell immunity. Along this line, interestingly, adult T-cell leukemia/lymphoma (ATLL) that developed in HTLV-1-infected T cells expresses high levels of IGSF4 on the cell surfaces, and IGSF4 expression may play a role in promoting cell-cell adhesion to the vascular endothelium, organ infiltration, and tumor growth in ATLL cells (37, 41).

Although the TCR is the key molecule that recognizes its cognate peptide and the MHC molecule, it does not induce intracellular signaling cascades without the assistance of various integral membrane proteins associated with TCRs. Thus, invariant chains, such as CD3γ,Δ,ϵ, and ζ-chains constitute the TCR-complex that initiates a series of intracellular signaling cascades (42). Additionally, co-receptors such as CD2, CD4, CD5, and CD8 and co-stimulatory molecules such as CD28 and LFA-1 play roles in controlling TCR signaling cascades (42). Lastly, membrane proteins such as TRAPs (TM adaptor proteins), LAT (linker for activation of T cells (43), TRIM (TCR-interacting molecule) (44, 45), PAG (protein associated with GEMs) (46), NTAL (non-T-cell activation linker) (47), LIME (LCK-interacting membrane protein) (48), and SIT [SH2-domain-containing protein tyrosine phosphatase (SHP2)–interacting TRAP] (49) have been identified to regulate TCR signaling. Among them, TRIM is also known to preferentially interact with the CD3 ζ-chain and elevate Ca^2+^ mobilization after T-cell activation through the regulation of TCR expression on the cell surface (44). However, transient or stable overexpression of IGSF4 does not affect TCR expression. Rather, it directly connects the CD3 ζ-chain to another ζ-chain (28) and controls the sizes of the TCR clusters, hence impacting TCR avidity.

The abilities of IGSF4 to form a self-dimer and, at the same time, bind to the CD3 ζ-chain may confer this protein’s interesting role. Many studies have reported that TCR clusters are observed during immunological synapse (IS) formation (50, 51). However, it has not been elucidated where and how the small TCR clusters form on the surfaces of cells. In addition, the stoichiometry of the TCR, which represents the number of copies of each subunit per complex, remains unresolved. This report suggests that the dimeric potency of IGSF4 can induce the multivalent stoichiometry of the TCR complex. In contrast, since the His177 residue in the IGSF4 TM region shares the Asp36 residue of the CD3 ζ-chain with the TCRα chain, it is also possible that IGSF4 can interfere with the TCR complex and lead to a decrease in TCR avidity. However, as deduced from the results obtained in the current study, in which the TCR avidity is increased, we think that the TCR complex is not tightly packed with each other but has a geometrical space for accommodating other TCR-associating molecules such as CD2, CD4, or CD8, and CD5 (52). If this is the case, however, the question is why the TCR clusters do not grow infinitely. A previous review suggested that TCR nanoclusters are accumulated in specific areas of the plasma membrane known as protein islands (16). However, recent studies by the present group (29) and Jung *et al*. (53) reported that the TCR complexes are located on the microvilli tips of resting T cells. These works suggest that microvilli form an important structural scaffold for TCR clustering. Therefore, the sizes of TCR clusters might be limited to the physical dimensions of the microvilli tips (54). The connection between TCR clusters and the actin bundles in microvilli may provide the mechanism by which TCR clusters become centrally accumulated through the cortical actin flow. Interestingly, a proteomic analysis of apical microvillus membranes revealed a high degree of similarity with lipid rafts (55).

The relationship between TCR strength and the antitumor response of cytotoxic T cells has been extensively studied, as this relationship is also closely coupled with autoimmunity (10, 39, 56–58). Indeed, since cancer cells are derived from normal cells of the body, the low immunogenicity of cancer antigens has often resulted in failure to control tumor growth. Nevertheless, incorrect high-affinity TCRs can also induce significant undesirable on-target/off-target destruction (12–15, 59). For this reason, several reports have demonstrated that moderate-affinity TCR has a better advantage for the antitumor response of T cells. Therefore, one way to increase the antitumor response is through the “functional avidity” of T cells—which refers to cellular responses in addition to binding—by stabilizing co-receptors or optimizing the signals generated by co-stimulatory molecules (39). Interestingly, a previous report demonstrated that high avidity CTLs can be elicited by exceedingly low concentrations of peptides (57), suggesting that the quality of CTLs is as important as the quantity in adoptive immunotherapy. Apart from enhancing functional avidity, however, there are no published studies that have attempted to selectively increase TCR avidity as a single factor. In the present study, the approach that utilized the property of the IGSF4 TM domain provides experimental evidence that directly shows whether or how TCR avidity affects the antitumor activity of T cells *in vivo*.

In activated T cells, TCR avidity is known to be enhanced *via* increased cross-linking of TCRs (22). Thus, the increase in the size of TCR pre-clusters due to the influence of IGSF4 can also induce constitutive activation of TCR signaling, which may increase the possibility of unexpected toxicity. Indeed, we observed slightly higher secretion of IFN-γ and TNF-α in CD8^+^ T cells expressing IG4ΔEXT in the absence of the antigen peptide (**Figure 5B**). Although a weak constitutive TCR activation is almost negligible in current animal tumor models, further studies will be necessary to determine what problems may occur in clinical applications (38).

In conclusion, the results of this study reveal that increased TCR avidity has a positive effect on TCR signaling and the subsequent T-cell activation. Further, this study provides experimental evidence that the avidity control of TCR as a single factor can increase the antitumor response of CTLs *in vivo*. Applications of this approach may be critical in developing effective adoptive T-cell immunotherapy for cancer as well as viral infections.

## Data Availability Statement

The original contributions presented in the study are included in the article/supplementary material. Further inquiries can be directed to the corresponding author.

## Ethics Statement

The animal study was reviewed and approved by Animal Care and Use Committee of the School of Life Sciences, Gwangju Institute of Science and Technology.

## Author Contributions

H-RK and J-SP wrote manuscript and created the figures. YF, MK, and J-HP performed the experiments. C-DJ wrote and finalized the review. All authors contributed to the article and approved the submitted version.

## Funding

This work was supported by the Creative Research Initiative Program (2015R1A3A2066253) through National Research Foundation (NRF) grants funded by the Ministry of Science and ICT (MSIT), the Basic Science Program (2019R1C1C1009570) through National Research Foundation (NRF) grants funded by the Ministry of Education (MOE), the National R&D Program for Cancer Control, Ministry for Health and Welfare (1911264), and supported by GIST Research Institute (GRI) IBBR grant funded by the GIST in 2020, Korea.

## Conflict of Interest

The authors declare that the research was conducted in the absence of any commercial or financial relationships that could be construed as a potential conflict of interest.
